# A bibliometric analysis of 30 years of research on transarterial radioembolization (TARE) for hepatocellular carcinoma

**DOI:** 10.3389/fphar.2024.1449722

**Published:** 2025-01-06

**Authors:** Tongyao Hu, Qifan Xu, Guorong Jia, Tao Wang, Changjing Zuo

**Affiliations:** ^1^ Department of Nuclear Medicine, The First Affiliated Hospital of Naval Medical University, Shanghai, China; ^2^ Department of Nuclear Medicine, No. 923 Hospital, Nanning, China; ^3^ School of Medical Imaging, Xuzhou Medical University, Xuzhou, China

**Keywords:** bibliometric analysis, hepatocellular carcinoma (HCC), transarterial radioembolization (TARE), ^90^Y microspheres, research trend, visualization

## Abstract

**Introduction:**

Patients with advanced hepatocellular carcinoma (HCC) have very limited treatment options, among which transarterial radioembolization (TARE) receives increasing attention, relying on its promising efficacy and fewer side effect. However, a bibliometric analysis of TARE for HCC is still lacking. This study employed bibliometric methods to analyze the related articles over the past three decades, and aimed to identify trends in clinical research comparing TARE to other treatments.

**Methods:**

Articles related with TARE for HCC were obtained from the Web of Science (WoS). After screening, the R package Bibliometrix was employed to explore the primary bibliometric characteristics. The number of publications was analyzed and mathematically fitted to a curve using Microsoft Excel 2021 and SPSS 25, and then was plotted in the graph using GraphPad Prism 8.0. VOSviewer, SCImago Graphica, and Pajek were utilized for the analysis of researchers’ co-authorship, co-occurrence, and visualization. Keywords citation burst was detected by CiteSpace software.

**Results:**

A total of 1,110 articles from 1993 to 2023 were included in our final analysis, among which the United States not only ranked the first in term of the number of published articles, also was at the forefront in other important indicators, including the total number of article citations and the average citation frequency. Riad Salem from Northwestern University, also being the organization with the greatest number of research papers, was the most active author and has published 96 papers. The keywords were classified into three clusters: ^90^Y microspheres for TARE, Basic research on TARE, and Clinical trial of TARE for HCC. Furthermore, we identified the most frequently cited keywords with strong citation bursts since 2020 were “multicenter,” “overall survival” and “PET/CT.”

**Conclusion:**

Our study employed a bibliometric approach to achieve the visualization research on TARE for HCC, and further revealed the trends and frontiers of TARE research, providing valuable information for researchers to identify the critical and persistent challenges and select potential partners in related area. Based on our analysis, future research focus include the clinical comparative studies on the effect of TARE and TACE combined with immunotherapies or targeted therapy, dosimetry, and personalized TARE therapy for HCC.

## 1 Introduction

With a relative 5-year survival rate of approximately 18%, hepatocellular carcinoma (HCC) is the sixth most common cancer and the third leading cause of cancer-related mortality worldwide (McGlynn et al., 2021; Sung et al., 2021). Currently, the HCC treatment options include liver resection, liver transplantation, ablation therapy, transarterial chemoembolization (TACE), transarterial radioembolization (TARE), and radiotherapy as well as systemic therapies (Vogel et al., 2022). Many patients with substantial intrahepatic tumor burdens, portal vein thrombosis (PVT), and declining liver function are diagnosed at an intermediate or advanced stage of the disease, and have no access to curative treatment. Hence, intra-arterial therapy is the preferred palliative treatment for candidates not eligible for resection or ablation, as hypervascular HCCs primarily receive blood supply from the hepatic artery. The most common intra-arterial therapy is TACE, which obstructs the tumor-feeding artery and causes ischemic necrosis of the tumors.

**FIGURE 1:**
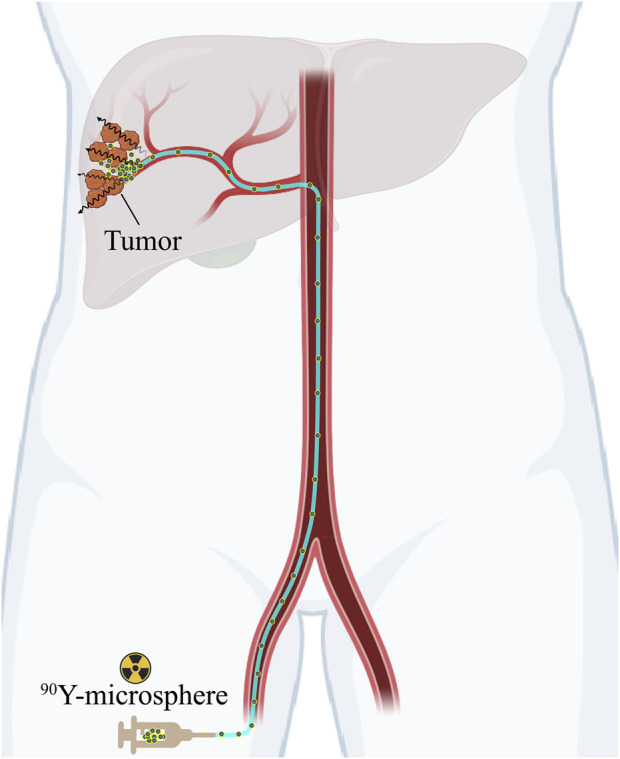
Schematic diagram of TARE with the clinical used ^90^Y-microspheres in the treatment of HCC.

Using the same technological technique as TACE, TARE, also known as selective internal radiation therapy (SIRT), involves hepatic artery intervention and therapeutic drug injections (Figure 1). However, TARE delivers radioactive microspheres without affecting hepatic arterial blood flow or causing ischemia. The advantage is that it increases the absorbed dose to the tumor while reducing systemic side effects and liver toxicity. TARE is a promising alternative therapy for unresectable HCC patients, as well as for patients with normal liver function but a high tumor burden or PVT. Hence, TARE serves as a locoregional palliative alternative or bridge therapy for liver transplantation in such patients. Studies have shown that treating patients with TARE results in high response and high survival rates as well as low toxicity (Levillain et al., 2021; Liu et al., 2022).

Bibliometrics is a widely used method that provides researchers with crucial insights into current research hotspots and relevant topics. It uses several analytical tools to visualize collaboration between countries, institutions, and authors. After identifying emerging developments or under-researched areas, it guides researchers’ research directions and gauges future research developments. In advancing clinical research, policymakers and funding agencies use bibliometric analyses to identify research priorities and allocate resources more efficiently to ensure that the most pressing clinical problems are addressed. Further identification of areas of insufficient research can be achieved through a process of comparison and analysis of the study’s results with the scientific issues present in clinical practice.

Although a lot of research has been done on TARE for HCC patients in the past three decades, a comprehensive and objective analysis depicting the current state of the art is still lacking. Thus, we used bibliometric methods to analyze the current trends of research on TARE for HCC patients and obtain an in-depth analysis of the relevant global development patterns. This study aimed to create a comprehensive knowledge map of academic publications on TARE for HCC, being profit to enhance readers’ comprehension and guide future research.

## 2 Methods

### 2.1 Data sources and search strategy

We chose Web of Science (WoS) as the target database because it is a common academic platform for scientific research and citation analysis, containing >12,000 of the world’s most authoritative and impactful academic journals and citation records. After obtaining relevant keywords and their supplementation with PubMed-sourced mesh subject headings, we conducted an exhaustive online bibliographic search with the following search queries: TS = (HCC OR “hepatocellular carcinoma*” OR “hepatic cell carcinoma*” OR “liver neoplasm*” OR hepatoma* OR “liver cell carcinoma*” OR “primary liver carcinoma*”) AND TS = (“Transarterial Radioembolization” OR Radioemboliz* OR Radioembolis* OR “Selective Internal Radiation Therapy”)—Time: March 6, 2024, 21:39:52 GMT+0800 (CST). The relevant articles were sourced from the beginning to December 31, 2023. Only “Article” was selected for the article type, and the language was limited to English. A thorough literature search yielded 2,259 potential records, of which we included 1,110 publications for analysis.

### 2.2 Data analysis and visualization

The WoS data was imported into Microsoft Excel 2021 for preliminary collation. We installed Bibliometrix package 4.1.4 in R 4.3.2 to extract bibliometric parameters like citation frequency as well as the number of publications based on year, country, and journal (Aria and Cuccurullo, 2017). GraphPad Prism 8.0 was used to draw the graphs.

VoSviewer 1.6.20 and SCImago Graphica 1.0.40 helped in the country cooperation network analysis. VOSviewer is a popular tool for constructing and visualizing bibliometric networks (van Eck and Waltman, 2010), while SCImago Graphica is a new tool for exploring and visually communicating data (Hassan-Montero et al., 2022). Additionally, VoSviewer 1.6.20 and Pajek 5.18, a commonly used package for analyzing and visualizing large networks (Batagelj and Mrvar, 2004), were used for keywords’ clustering and co-occurrence network analyses, respectively.

CiteSpace is a software tool, used for citation analysis and visualization. It helps in visualizing the structure, distribution, and trends of academic information through a process known as “scientific knowledge mapping” (Synnestvedt et al., 2005). CiteSpace’s citation burst analysis extracts keywords or references that have significantly changed over time and can reveal novel research hotspots from different perspectives. Citation intensity and burst time are the indicators that evaluate the strength of attractiveness and the activity time of the keyword or reference, respectively. Thus, we used CiteSpace to identify highly cited references/keywords that experienced strong citation bursts in a specific period.

Our study’s primary data were obtained from public databases and did not require an ethical review.

## 3 Results

### 3.1 Search results and study selection

**FIGURE 2:**
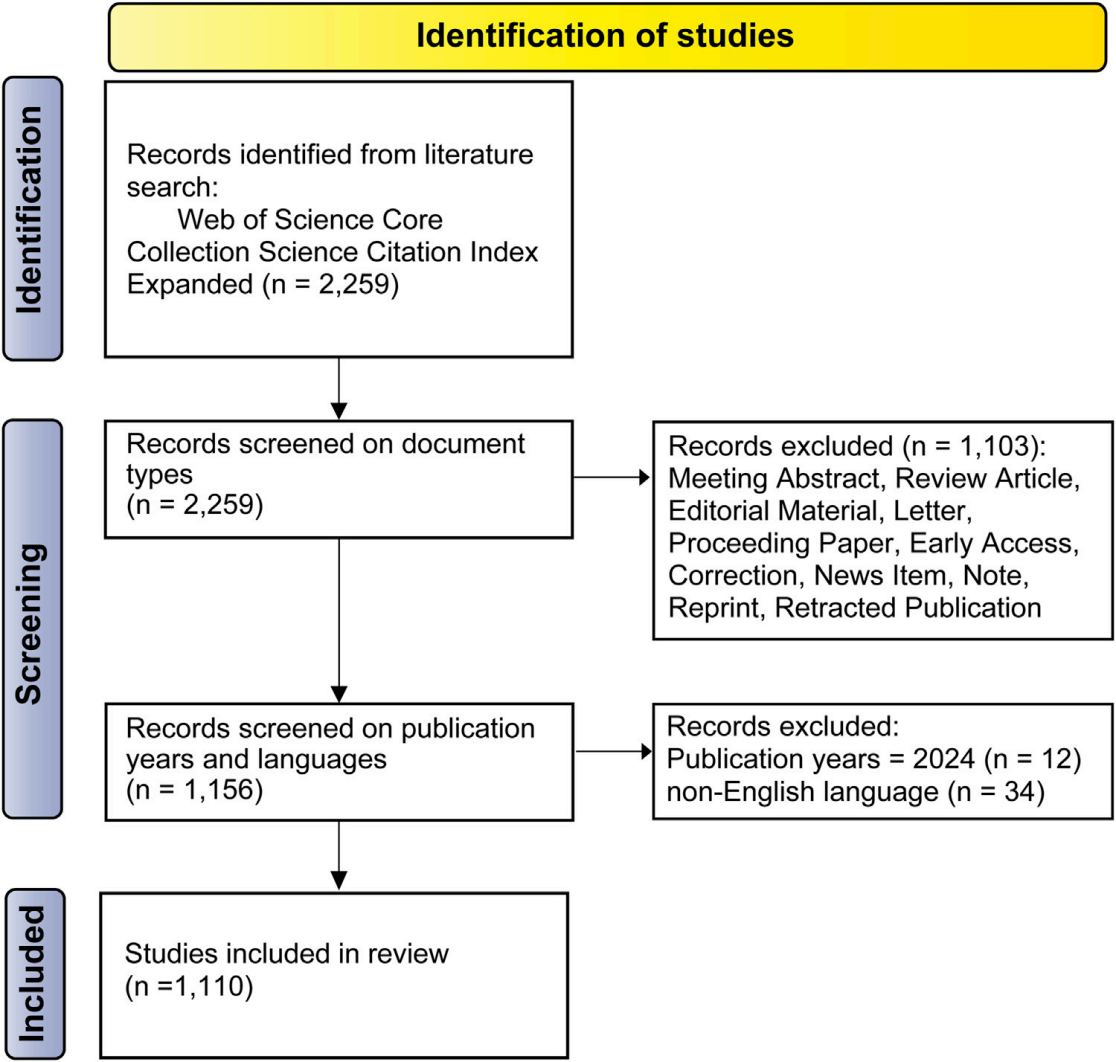
Flow chart depicting literature search, screening as well as the study identification process, adapted from the PRISMA statement.

The literature search yielded 2,259 results, and 1,110 articles were included for analysis post-screening (Figure 2). Based on the WoS data, 1,110 publications had amassed a total of 37,851 citations. With an H-index of 83, the mean citation frequency per article was 34.1 citations.

### 3.2 Annual publications trends

**FIGURE 3:**
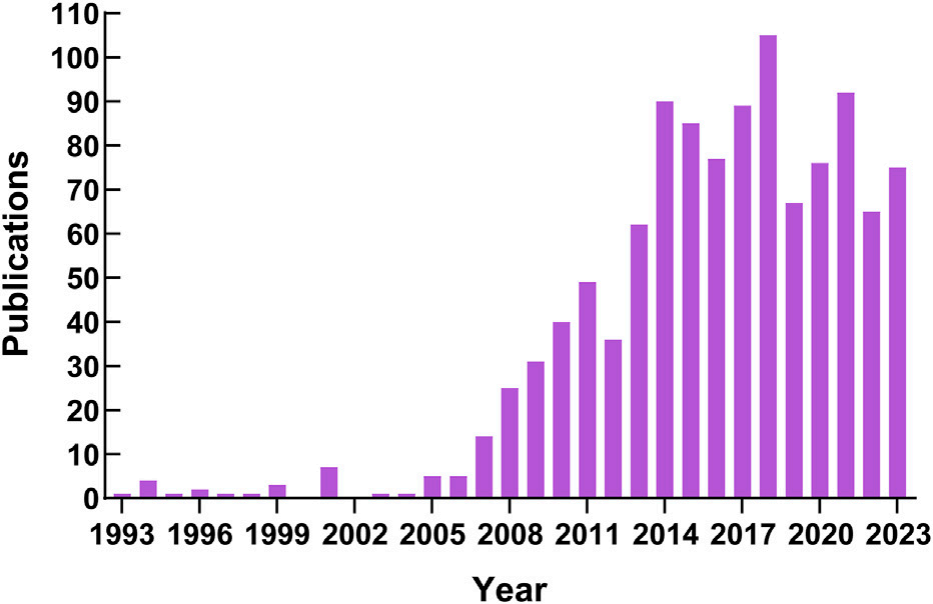
Annual publications trends of TARE research on HCC.

Figure 3 shows the TARE articles’ annual publication volume for HCC. The earliest article was published in 1993. From 1993 to 2006, the annual publication number was <10, with some years providing no contributions (2000 and 2002). Moreover, the number of annual publications exhibited a gradual upward trend with occasional minor fluctuations from 2007 to 2018. However, the publication number declined but stabilized after 2018.

### 3.3 National publication volume and collaboration

A national publication analysis revealed that 34 countries/regions had published relevant articles. Based on the corresponding author, Table 1 displays the top ten productive countries, which accounted for 86.13% of all publications. Among them, the United States of America (United States) contributed the maximum publications (n = 445), accounting for 40.09% of all publications. The US was also leading in other key metrics, such as total and average citations. Germany ranked second (n = 132, 11.89%), followed by France (n = 75, 6.76%), Italy (n = 71, 6.40%), and China (n = 67, 6.04%). The remaining countries had <60 publications.

**TABLE 1:** The top ten productive countries based on the corresponding author’s information.

Countries	N	Single country publications (SCP)	Multiple country publications (MCP)	Total citations	Average citations	%	H-index
USA	445	387	58	17,536	39.41	40.09	59
Germany	132	106	26	3386	25.65	11.89	32
France	75	64	11	3160	42.13	6.76	26
Italy	71	56	15	2234	31.46	6.40	22
China	67	63	4	1135	16.94	6.04	18
Korea	52	47	5	578	11.12	4.68	16
Spain	35	26	9	2938	83.94	3.15	16
Netherlands	31	23	8	869	28.03	2.79	17
Belgium	25	20	5	391	15.64	2.25	15
Switzerland	23	19	4	480	20.87	2.07	14

**FIGURE 4:**
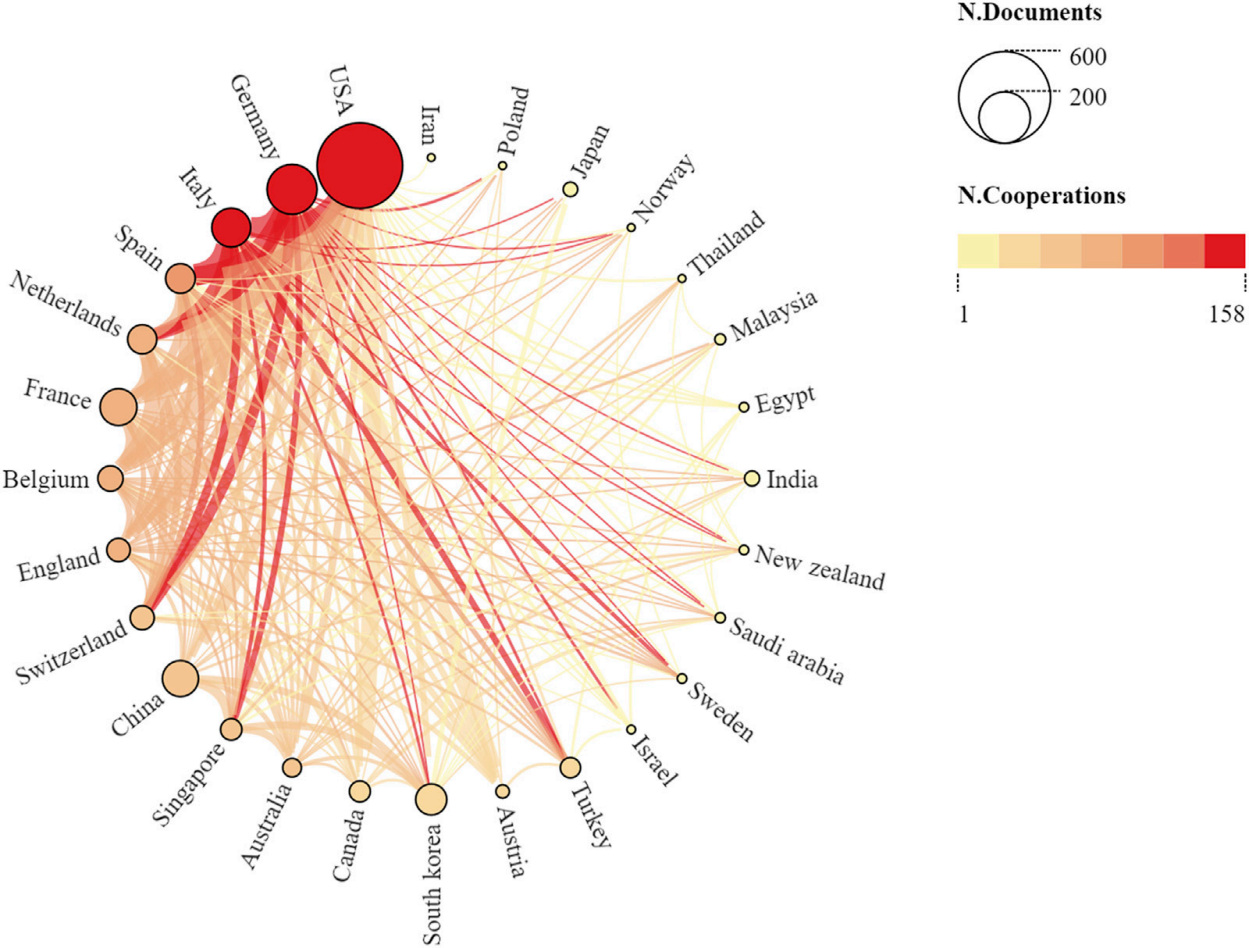
Comparative map showing the cumulative publication and cooperation networks across the country. While nodes represent countries, each node’s size is proportional to the number of publications. Based on the number of collaborations, the nodes are arranged in counter-clockwise order and a link between two nodes represents a collaboration. The link’s width indicates the strength of a specific collaboration. Moreover, if the two countries cooperated more closely, the link between them became thicker and hotter.

Multiple country publications (MCP) revealed academic collaborations between different countries and geographical regions. Figure 4 presents a global perspective of publications and international collaborations in this field. The US, Germany, and Italy ranked as the top three countries in terms of international cooperation. Despite being among the top ten productive countries, China and South Korea provided insufficient global cooperation. Therefore, for these countries, more efforts should be made to strengthen international cooperation and communication.

### 3.4 Institutional output and collaboration

We included a total of 1,374 institutions in the related research of TARE for HCC. Table 2 lists the top ten productive institutions, with Northwestern University ranking first with a maximum of 346 publications. Of these, the US had three institutions, while Germany and South Korea had two each. Singapore, Spain, and the Netherlands each had one institution.

**TABLE 2:** The top ten productive institutions.

Affiliation	Country	Articles
NORTHWESTERN UNIV	United States	346
ROBERT H LURIE COMPREHENS CANC CTR	United States	79
SEOUL NATL UNIV	Korea	77
UNIV HOSP ESSEN	Germany	77
SINGAPORE GEN HOSP	Singapore	71
UNIV NAVARRA CLIN	Spain	69
YONSEI UNIV	Korea	68
UNIV MUNICH	Germany	66
ICAHN SCH MED MT SINAI	USA	65
UNIV MED CTR UTRECHT	Netherlands	65

**FIGURE 5:**
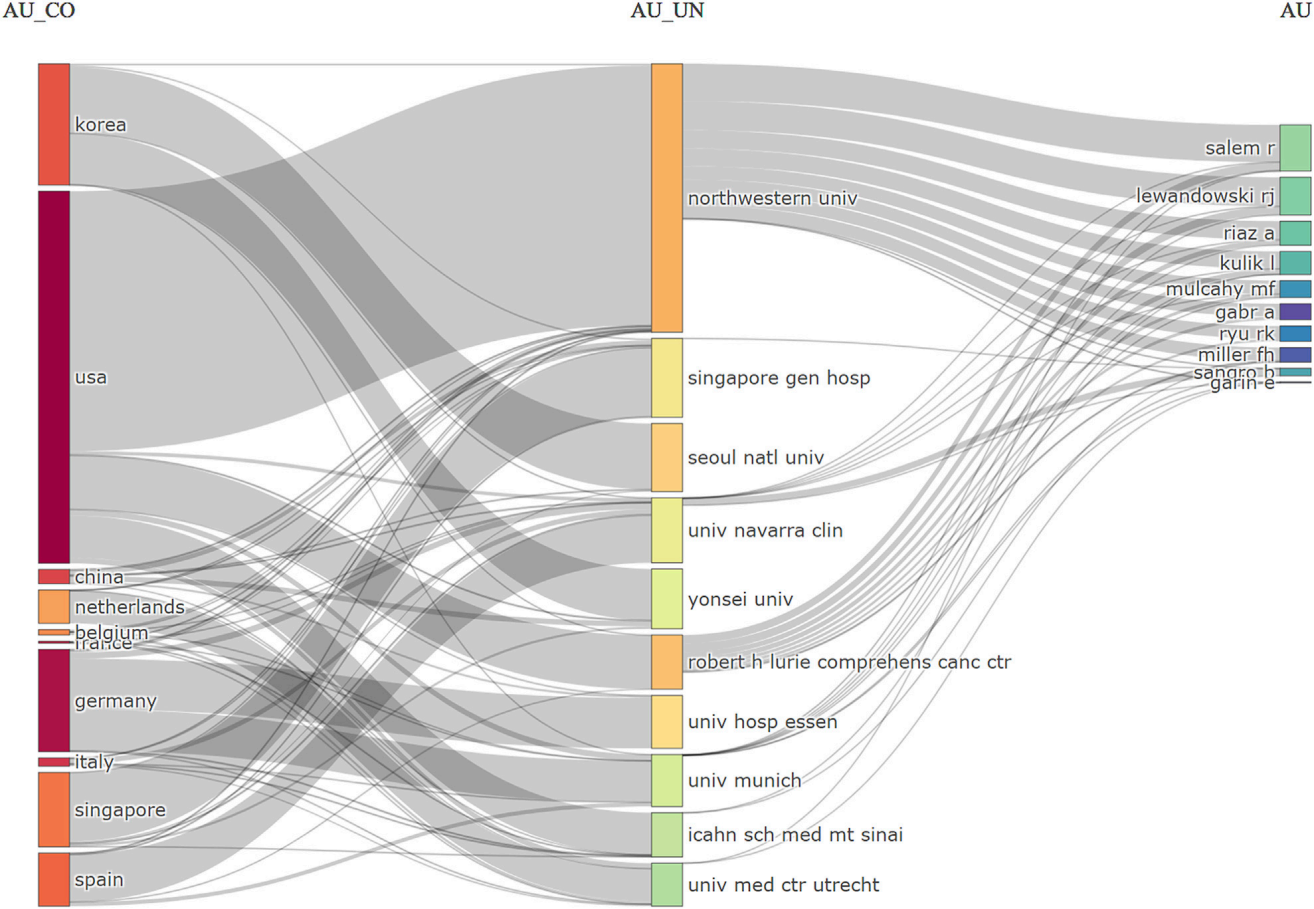
The three-field plot (Country-Affiliation-Author).

A three-field plot was utilized to map collaborative networks to investigate the collaborative relationships among the leading countries, institutes, and authors (Figure 5). Northwestern University, United States, was the leading institute, with Prof. Riad Salem as its central figure. The most productive authors collaborated more closely than others.

### 3.5 High-contributing journals

A total of 262 journals had published relevant articles. Table 3 shows that the top ten journals in terms of publication volume published 387 papers (34.86%), with 6 and 3 from the United States and Germany, respectively. Among them, *the Journal of Vascular and Interventional Radiology* had the highest number of publications (111), total citations (3700), and H-indexes (47), respectively. *The International Journal of Radiation Oncology Biology Physics* had the highest average citations (103.72). Of these ten journals, four each were classified in Journal Citation Reports (JCR) Quartile 1 (Q1), and JCR Q2, respectively.

**TABLE 3:** The top ten productive journals.

Journal	Country	N	Total citations	Average citations	H-index	IF-2022	JCR
Journal of Vascular and Interventional Radiology	United States	111	3700	33.33	37	2.9	Q2
CardioVascular and Interventional Radiology	United States	64	1447	22.61	20	2.9	Q2
Journal of Nuclear Medicine	United States	44	2011	45.70	25	9.3	Q1
European Journal of Nuclear Medicine and Molecular Imaging	Germany	36	1453	40.36	23	9.1	Q1
Nuclear Medicine Communications	United States	28	289	10.32	9	1.5	Q4
Seminars in Interventional Radiology	United States	26	94	3.62	5	1.4	Q4
Cancers	Switzerland	22	139	6.32	7	5.2	Q2
EJNMMI Research	Germany	19	406	21.37	9	3.2	Q2
European Radiology	Germany	19	638	33.58	13	5.9	Q1
International Journal of Radiation Oncology Biology Physics	United States	18	1867	103.72	14	7.0	Q1

### 3.6 Funding agencies

Table 4 lists the top ten funding agencies. Ranking first with 6.76%, the United States Department of Health and Human Services supported 75 studies. Among the top funding organizations, three were from the United States, and one each was from China, Germany, France, and South Korea, respectively. The remaining three were pharmaceutical companies. Thus, appropriate funding, particularly government funding, should be increased for this research.

**TABLE 4:** The top ten funding agencies.

Funding agency	N	%
United States Department of Health Human Services	75	6.76
National Institutes of Health NIH USA	74	6.67
Bayer AG	48	4.32
Sirtex Medical	47	4.23
NIH National Cancer Institute NCI	26	2.34
National Natural Science Foundation of China NSFC	13	1.17
German Research Foundation DFG	8	0.72
Siemens AG	8	0.72
Agence nationale de la recherche ANR	7	0.63
National Research Foundation of Korea	7	0.63

### 3.7 Author collaboration network graph

A total of 5,356 authors participated in the TARE-related studies for HCC. Table 5 displays that Prof. Riad Salem was the most productive author with 96 publications (Local H-index = 54), followed by Robert Lewandowski and Ahsun Riaz with 75 (Local H-index = 42), and 45 publications (H-index = 28), respectively. Notably, seven out of ten authors were from Northwestern University.

**TABLE 5:** The top ten productive authors in the related studies of TARE for HCC.

Authors	Articles	Local H-index	Institution and country
Riad Salem	96	54	Northwestern University,United States
Robert Lewandowski	75	42	Northwestern University,United States
Ahsun Riaz	45	28	Northwestern University,United States
Laura M Kulik	41	29	Northwestern University,United States
Bruno Sangro	35	19	Clinica Universidad de Navarra, Spain
Mary F Mulcahy	33	27	Northwestern University,United States
Robert K Ryu	31	23	University of Colorado School of Medicine,United States
Etienne Garin	30	19	Centre Eugène Marquis, France
Frank H Miller	26	22	Northwestern University,United States
GABR A	25	15	Northwestern University,United States

Eleven clusters were created using VoSviewer to generate author collaboration network graphs from 247 authors with >5 published articles, as seen in Supplementary Figure S1. Cluster 1 primarily comprised Northwestern University researchers, with Riad Salem as the key researcher. The core figure of Cluster 3 was Bruno Sangro, affiliated with Clinica Universidad de Navarra. However, these clusters did not collaborate much. Supplementary Figure S2 depicts the time-overlapping map for co-authorship analysis among 247 researchers. Moreover, Hyo-Cheol Kim of Seoul National University College of Medicine has been actively involved in TARE-related work for HCC in recent years.

### 3.8 Characteristics of the top ten most cited publications

Highly cited papers can provide significant insights regarding the development of academic impact. Analyzing the most cited publications helps beginners understand the progress of specific research topics. Moreover, it can also provide a basis for subsequent in-depth or systematic research explorations in response to previous results. Table 6 displays the ten most cited articles, which were cited 4,647 times and represented 12.28% of all publications. With 741 citations, the most cited article was authored by Riad Salem, with an annual average of 49.4 citations. The article with the highest average annual citation count of 66.75 was published by Valérie Vilgrain. The use of ^90^Y microspheres was the topic of the ten most cited articles. Of these, seven and three articles used ^90^Y glass microspheres (TheraSpheres) and ^90^Y resin microspheres (SIR-Spheres), respectively. Notably, multi-center, randomized controlled trials receive more citations.

**TABLE 6:** The top ten frequently cited papers.

No	First author	Journal	Year	Citations	Citation frequency per year	Descriptions
1	Riad Salem	GASTROENTEROLOGY	2010	741	49.40	This single-centre, prospective, and longitudinal study reported the long-term safety and response rates of TheraSpheres in TARE-related studies for HCC. The survival outcomes of TARE and TACE were similar. The main survival predictors were liver function and PVT. Moreover, patients with Child-Pugh A disease, with or without PVT, benefited most from this treatment
2	Valérie Vilgrain	LANCET ONCOL	2017	534	66.75	SARAH was a multi-centre, open-label, and randomized controlled phase 3 trial comparing the efficacy and safety of SIR-Sphere SIRT with sorafenib. No significant difference was observed in overall survival between the two groups with locally advanced or intermediate-stage HCC after an unsuccessful TACE. However, the SIRT group exhibited enhanced tumour response, quality of life, and safety than the sorafenib group
3	Bruno Sangro	HEPATOLOGY	2011	488	34.86	This retrospective multi-centre analysis evaluated the survival prognostic factors after SIR-Sphere TARE for HCC. Liver function and Child-Pugh scores were significant survival predictors. Moreover, TARE might achieve survival benefits across different tumour stages, advanced disease patients, and those with few treatment options
4	Riad Salem	GASTROENTEROLOGY	2011	478	34.14	This retrospective study compared the efficacy of TACE with TheraSphere TARE in HCC patients. Both these therapies provided similar survival outcomes; however, TARE was better tolerated than TACE, with fewer toxicities and without any inpatient hospitalization. The study indicated that a randomized trial with >1000 patients can establish the equivalence of survival times between patients treated with TARE and TACE.
5	Laura M Kulik	HEPATOLOGY	2008	458	26.94	This prospective phase 2 cohort study revealed the safety profile and efficacy of TheraSphere TARE for unresectable HCC, with or without PVT. Thus, TARE is a safe procedure with favourable tumour response rates for HCC patients complicated by branch/lobar PVT.
6	R J Lewandowski	AM J TRANSPLANT	2009	427	26.69	This single-centre study compared the downstaging efficacy of TACE and TARE. Although both treatments significantly reduced the tumour size from baseline, TheraSphere TARE outperformed TACE for downstaging HCC from UNOS T3 to T2
7	Pierce K H Chow	J CLIN ONCOL	2018	415	59.29	This multi-centre, open-label, phase 3 trial compared the efficacy of SIR-Sphere TARE and sorafenib in locally advanced HCC patients. Overall survival was similar between TARE and sorafenib in locally advanced HCC patients. However, TARE displayed a better tumour response rate and fewer adverse events than sorafenib
8	Riad Salem	GASTROENTEROLOGY	2016	403	44.78	This randomized, phase 2 study compared the effectiveness of cTACE and TheraSphere TARE in HCC patients of BCLC stages A or B. The results showed that TARE provided a significantly longer time to progression and reduced transplant waitlist dropout rates
9	Vincenzo Mazzaferro	HEPATOLOGY	2013	363	30.25	This prospective phase 2 study evaluated the efficacy and safety of TheraSphere TARE in intermediate and advanced HCC. The study suggested that TARE was effective in intermediate to advanced HCC cases, particularly in PVT patients
10	Philip Hilgard	HEPATOLOGY	2010	340	22.67	This single-centre observational study evaluated the efficacy of TheraSphere TARE in European advanced HCC patients and showed that TARE was a safe and effective treatment for European HCC patients and could be used in impaired liver function patients

### 3.9 Keyword analysis of global research

**FIGURE 6:**
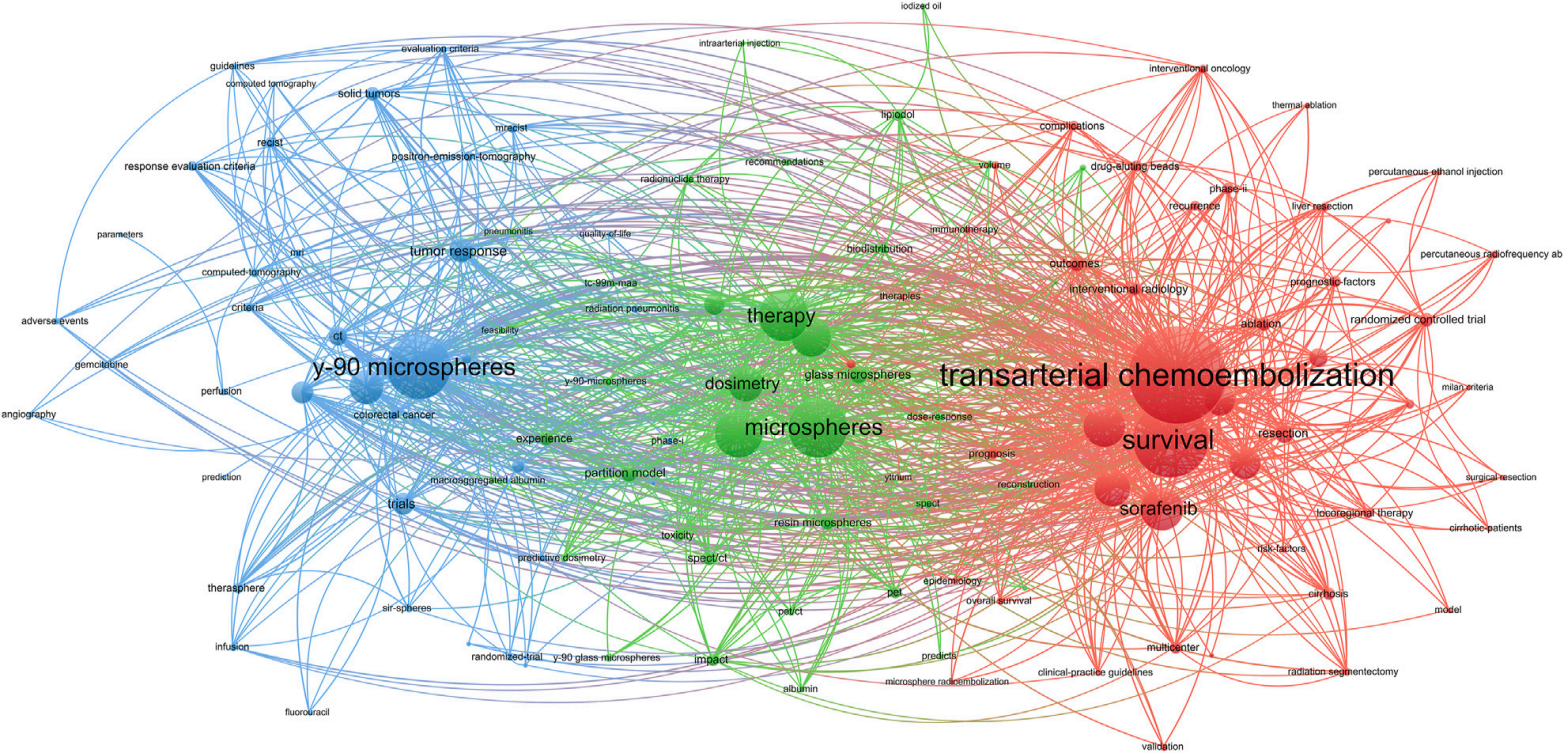
The keyword co-occurrence network. Each node’s size is proportional to the number of papers containing the corresponding keyword. The keywords that appear together in an article are represented as an edge linking two nodes. The edge’s width indicates the number of articles where the keywords co-occur, and the colour reflects the keyword cluster. More closely associated keywords are classified into the same cluster.

**FIGURE 7:**
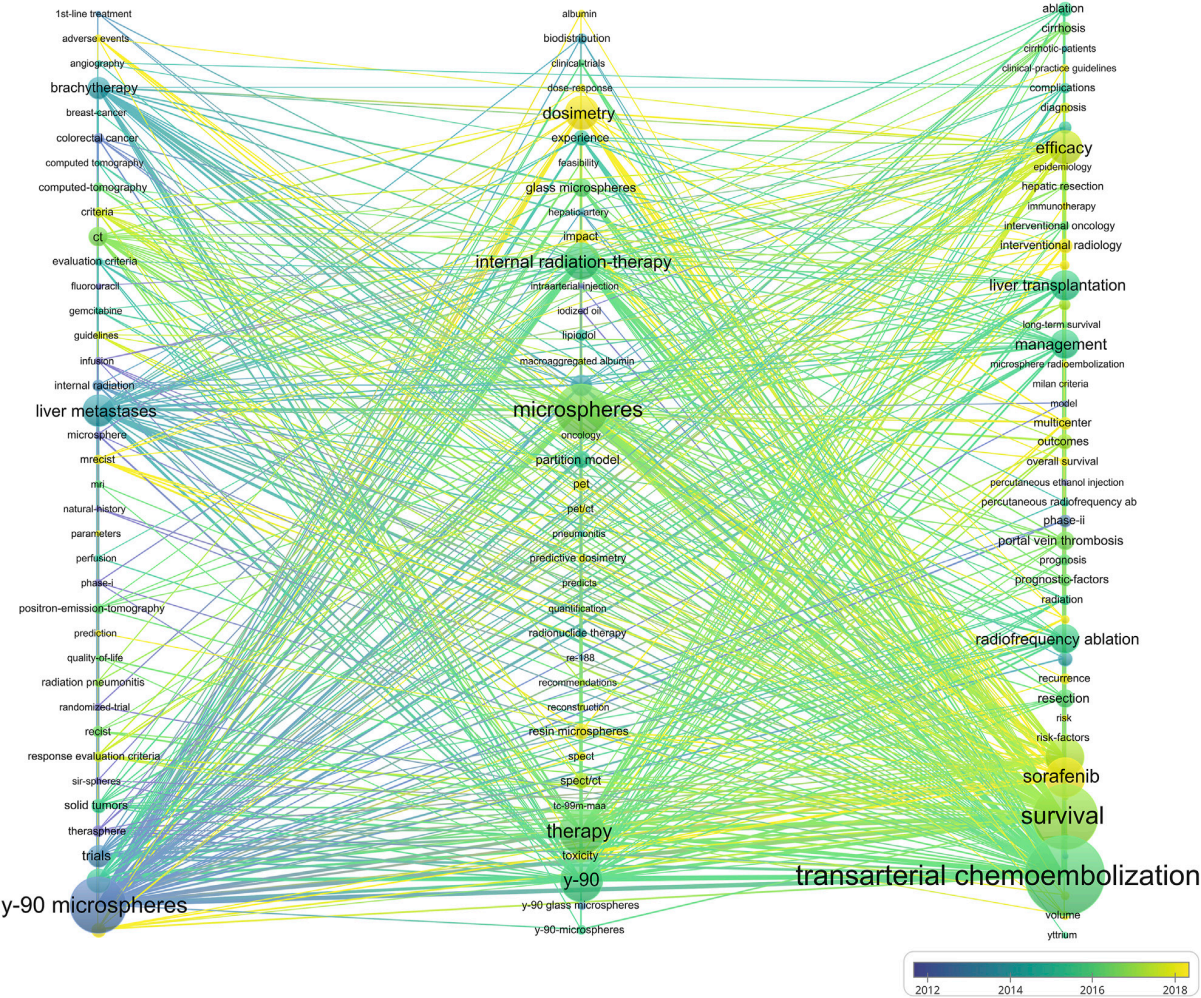
Keyword co-occurrence plus time-overlapping network.

After filtering keywords, 238 words were included in the co-occurrence analysis (Figure 6). We divided the keywords into 3 clusters: “^90^Y microspheres for TARE” (Cluster 1, blue), “Basic research on TARE” (Cluster 2, blue), and “Clinical trial of TARE for HCC” (Cluster 3, red). Cluster 1 was colored blue and focused on using ^90^Y microspheres for TARE. The primary keyword was “^90^Y microspheres,” with emphasis on “liver metastases” and “brachytherapy.” Cluster 2 was green in color and focused on basic TARE-related research. The primary keyword was “microsphere,” with others, including “dosimetry “and “toxicity.” Cluster 3 was the largest and red in color, primarily focused on clinical trials and the primary efficacy outcome of “survival.” It also compared TARE with TACE and sorafenib. Figure 7 displays the time-overlapping analysis network of co-occurring keywords, with colors ranging from dark blue to light yellow that represent the keywords’ average active years. The interests of researchers’ investigations gradually shifted from Cluster 1 to Clusters 2 and 3, respectively.

### 3.10 Citation burst analysis

**FIGURE 8:**
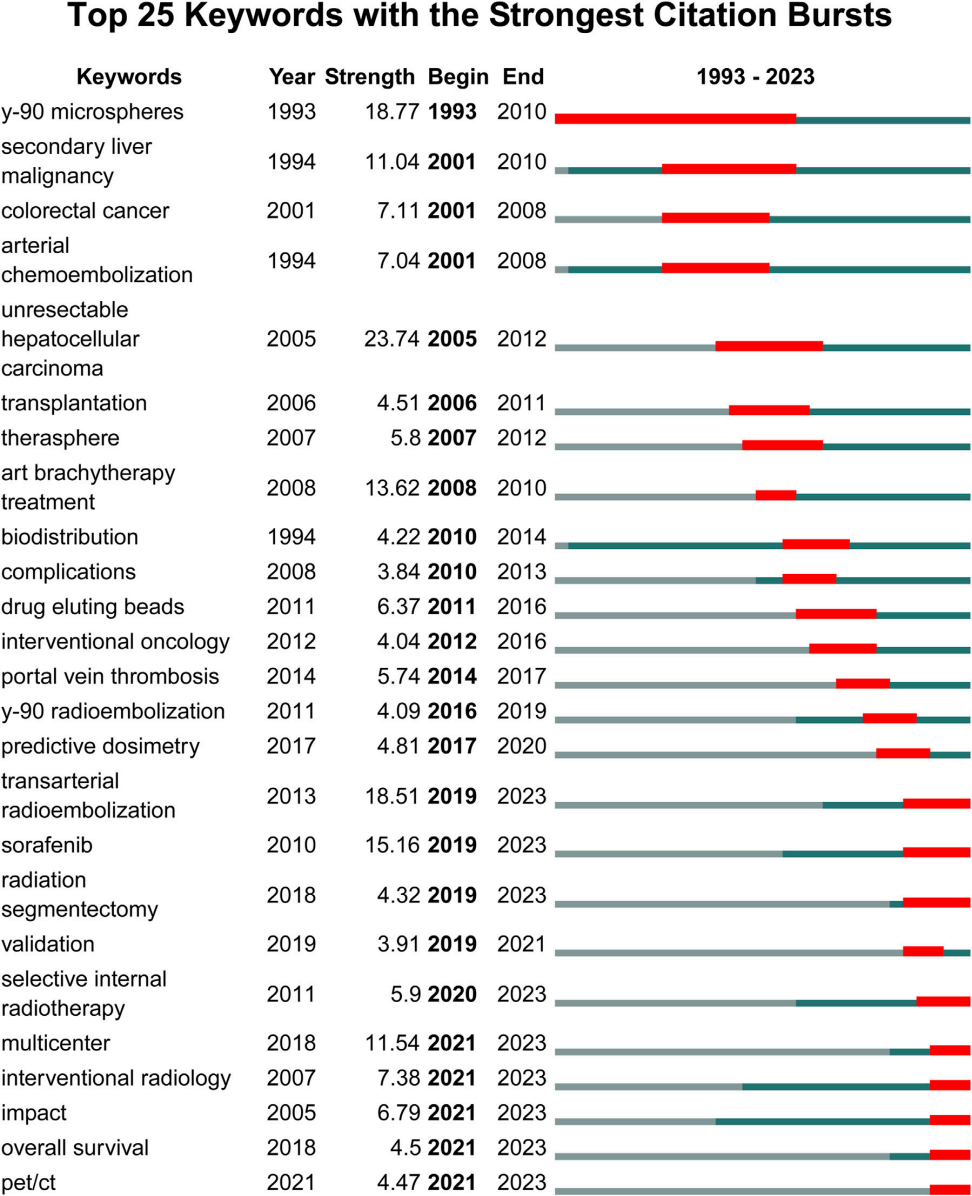
The top 25 keywords with robust citation bursts.

**FIGURE 9:**
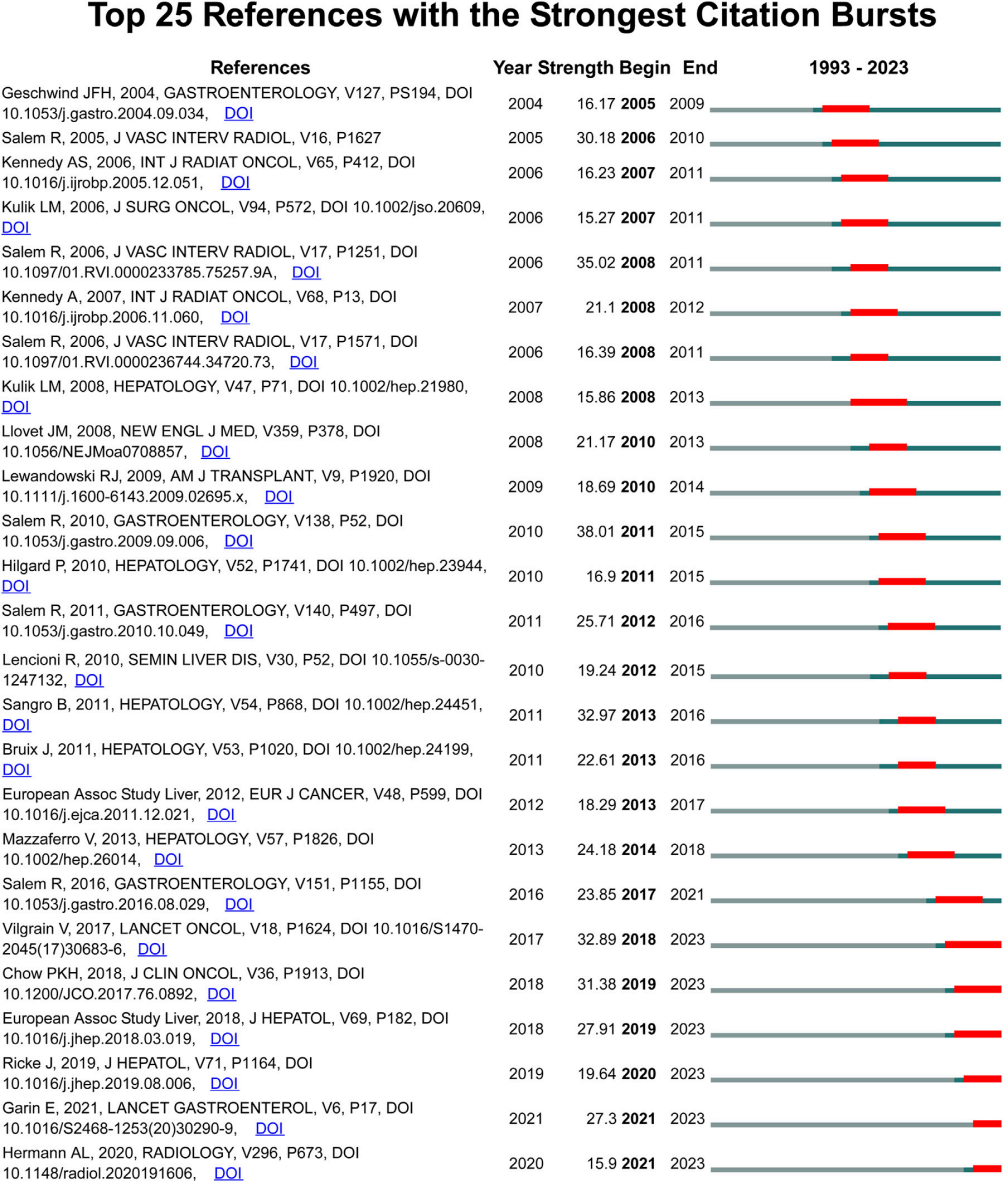
The top 25 references with the strongest citation bursts.

Although VoSviewer is useful for displaying keyword co-occurrence, it is less effective in illustrating changes in keyword prominence. It merely visualizes annual publication activity and does not indicate the start as well as end times or keywords’ sudden bursts. Hence, we used CiteSpace software to extract citation bursts for all keywords, with emphasis on the top 25 keywords. However, keywords like “multi-center,” “overall survival,” and “PET/CT,” which suggest promising developments are more in need since 2020 (Figure 8). Citation bursts were conducted for the references; Figure 9 displays the top 25 references with the strongest citation bursts. In this study, the keywords “sorafenib,” “multi-center,” and “overall survival” had the latest burst till 2022. Moreover, these keywords were also identified in the time-overlapping and keyword analyses, respectively.

## 4 Discussion

In 2018, Miao Yan et al. conducted a bibliometric analysis of 24,331 papers on HCC published between 2008 and 2017 (Miao et al., 2018), and found that, the overall trend of the annual publications has consistently increased from 1,348 articles in 2008 to 3,572 articles in 2017. China has contributed the greatest number of publications, while the United States has been the leader in the H-index and the ESI top papers. The results of the citation bursts analysis indicated that the keyword “transarterial chemoembolization” was the most prominent emerging topic in 2017.

In Das et al. (2020) conducted a bibliometric analysis of the top 100 most cited articles from 5,873 papers on TACE for HCC, and their results demonstrated that Japan published the highest number of highly cited papers (24), followed by the United States (14) and China (14). Among the top 100 articles on TACE for HCC, the journal *Cancer* (16), *Radiology* (13) and *Hepatology* (11) published the most articles. The most prevalent primary focus of the top 100 articles was conventional TACE monotherapy, whereas TARE was the most common primary focus of conventional TACE combination therapy.

In 2023, Na Zhang et al. conducted a bibliometric analysis of 5728 papers on TACE for HCC published between 2012 and 2021 (Zhang et al., 2023). The number of papers published annually has exhibited a consistent upward trajectory. China has the highest number of papers, while the United States has been the leader in terms of the H index. *The Journal of Vascular and Interventional Radiology* was the most prolific publication in the field, with 234 articles. The keyword co-occurrence analysis identified the research topic “prediction of TACE treatment” as the most significant topic of interest.

Unlike the published bibliometric studies mentioned above, this study analyzed publications on TARE for HCC patients from 1993 to 2023 using the bibliometric analysis method. Yan et al. (1993) discussed the effectiveness of internal radiation therapy using TheraSpheres administered through the portal vein as a non-surgical treatment for HCC. Based on the number of annual publications, the publication growth trends can be divided into three phases: the initial, growth, and stable phases, respectively. From 1993 to 2006, the initial period saw <10 publications per year. The growth period from 2007 to 2018 demonstrated an overall upward trend in the number of publications per year. However, from 2019 to 2023, all the relevant studies entered a stable phase having >60 annual publications. This suggests that the research on TARE for HCC will remain active in the coming years because of poor prognosis and limited treatment options for intermediate and advanced HCC patients (Salem, 2023). Hence, more and more organizations and countries are prioritizing and investing in this area to enhance the safety and efficacy of treatments for HCC as well as to improve the life quality and survival rate. Annually, approximately 50% of the new HCC cases and HCC-related deaths occurs in China (Rumgay et al., 2022). In 2022, SIR-Sphere TARE was approved in China for HCC patients. Predictably, patients worldwide will have access to TARE as more clinical trials progress to determine the efficacy and safety of TARE for Asian HCC patients.

The USA has the highest number of HCC publications and citations in the field of TARE. It is a major contributor to international collaborations, representing the global frontier level. Thus, the majority of the top 10 institutions, authors, and journals all are from the USA. This might be due to the emphasis and substantial funding of the USA healthcare field, leading to the development of TARE for HCC treatment.

Among the top ten productive journals, only four have published >30 research articles on TARE for HCC. The two journals, including *Journal of Vascular and Interventional Radiology* and *CardioVascular and Interventional Radiology*, have the maximum publications among them. Two classic nuclear medicine journals, the *Journal of Nuclear Medicine* as well as the *European Journal of Nuclear Medicine and Molecular Imaging*, are second in publications. These core journals publish core research within the field and represent cutting-edge advances in this discipline. Therefore, researchers should submit their manuscripts to top journals in the interventional and nuclear medicine field. Although China and South Korea are also active in publications, none of the top ten journals are published by an Asian publisher. This is because of the delayed relevant research initiation in Asian countries. However, the analyses suggest that China and South Korea have the potential to create international impact journals.

The top ten most cited articles were all clinical studies on ^90^Y microspheres, suggesting that high-quality clinical studies receive a large number of citations. Of these, seven and three articles were on TheraSpheres and SIR-Spheres, respectively. Currently, TheraSphere and SIR-Spheres are the two FDA-approved microspheres for the treatment of HCC. These two microspheres are different in terms of their production methods, physical properties, and methods of use. The trial’s primary outcomes were safety and response rate, with the main focus on comparisons of TARE with other treatments like TACE and sorafenib. The ten most cited articles focused on the following topics: “PVT,” “liver function,” “survival,” “toxicity,” “TACE,” “sorafenib,” “multicenter,” and “downstaging.”

Keywords are an integral part, and their frequency indicates their influence in a particular field. This study categorized keywords into three groups. Cluster 1 focused on the ^90^Y microsphere usage for TARE. ^90^Y is currently the most widely used radionuclide for the clinical application of TARE for HCC. Its advantages include the emission of pure (100%), high-energy (average energy 932.9 keV, maximum energy 2.28 MeV) β-rays with weak soft-tissue penetration (maximum of about 12 mm), and moderate physical half-life (64 h). Currently, other radionuclide microspheres are either in the preclinical or basic research stages. Preliminary evidence has demonstrated the efficacy and safety of ^166^Ho microsphere TARE in unresectable and chemotherapy-resistant liver metastases.

Cluster 2 focused on basic TARE research, especially for microspheres. Advances in materials science lead to innovations in drug delivery, and nanomaterials, liposomal molecules, and other delivery systems can serve as new carriers to improve the efficacy of TARE. Novel degradable materials can be used as carriers to boost the effectiveness of TARE (Shi et al., 2023). Combining radionuclides with other drug delivery systems like nucleic acids and microrobots contribute to decrease the radiotoxicity and immunogenicity of radionuclide therapy, leading to improved targeting of TARE (Nelson and Pané, 2023). Besides, molecular biology and immunology advancements might identify specific HCC targets, and further enhance the specificity of radionuclide therapy’s (Calderaro et al., 2017). Hence, future research should focus on appropriate dosimetry and toxicity reduction of various TARE microspheres.

The largest group, Group 3 focused primarily on clinical trials, and its keyword was associated with the primary efficacy outcome of survival. Therefore, future studies should focus on large, multi-center trials to compare TARE with other treatments in HCC patients with varied liver function status. In particular, research should focus on exploring the potential synergistic effects of combining TARE and TACE with immunotherapy and targeted therapies.

As for references, the burst of the six most cited publications has remained consistent until 2023. Of them, three focused on the comparison between TARE and sorafenib (Vilgrain et al., 2017; Chow et al., 2018; Ricke et al., 2019) while the two explored the personalized dosimetry and tumor radiation–absorbed doses (Hermann et al., 2020; Garin et al., 2021). The remaining study was the European Association for the Study of the Liver (EASL) updated HCC guideline and evaluated the efficacy of TARE (EASL and European Association for the Study of the Liver, 2018). The reference hotspots aligned with the keyword hotspots.

This study also has several limitations. Firstly, we only included English-language research articles from the WoS database and may omitted some relevant studies. Since WoS includes the majority of high-impact journals, we infer that this omission does not affect the field’s overall trend. Secondly, the dependence of bibliometric analysis on the software used for analyzing and processing the data might introduce some bias. In contrast to systematic reviews, it permits a comprehensive analysis of extensive data. This outlines the field’s overall picture and provides the researcher with clues to current research from different perspectives. Lastly, the annual publications’ mathematical prediction model is based solely on past data, but in reality, the relevant publications’ growth trend is even more robust.

## 5 Conclusion

In conclusion, TARE treatment for HCC has attracted considerable attention, and research in this area is ongoing. Based on our analysis, future research topics might include: large, multi-center, and real-world studies comparing TARE and TACE with immunotherapy and targeted therapies in HCC patients with different liver function grades; evaluating the personalized dosimetry and toxicity of ^90^Y microspheres as well as developing novel radioembolization devices, microsphere materials, and radionuclides to increase the absorbed radiation dose to tumors and reduce toxicity. This study will help researchers understand the trends, hotspots, and frontiers in the related research of TARE for HCC. With our results, researchers might be well-equipped to conduct a more accurate and extensive study in this area.

## Data Availability

The original contributions presented in the study are included in the article/Supplementary Material, further inquiries can be directed to the corresponding authors.

## References

[B1] Aria M. Cuccurullo C. (2017). bibliometrix: an R-tool for comprehensive science mapping analysis. J. Inf. 11 (4), 959–975. 10.1016/j.joi.2017.08.007

[B3] Batagelj V. Mrvar A. (2004). “Pajek — analysis and visualization of large networks,” in Graph drawing software. Editors Jünger, M. Mutzel P. (Berlin, Heidelberg: Springer Berlin Heidelberg), 77–103.

[B4] Calderaro J. Couchy G. Imbeaud S. Amaddeo G. Letouzé E. Blanc J. F. (2017). Histological subtypes of hepatocellular carcinoma are related to gene mutations and molecular tumour classification. J. Hepatol. 67 (4), 727–738. 10.1016/j.jhep.2017.05.014 28532995

[B5] Chow P. K. H. Gandhi M. Tan S. B. Khin M. W. Khasbazar A. Ong J. (2018). SIRveNIB: selective internal radiation therapy versus sorafenib in asia-pacific patients with hepatocellular carcinoma. J. Clin. Oncol. 36 (19), 1913–1921. 10.1200/jco.2017.76.0892 29498924

[B6] Das J. P. Thulasidasan N. Ahmed I. Diamantopoulos A. (2020). Transarterial chemoembolization for hepatocellular carcinoma: a bibliometric analysis of the most cited articles. Jpn. J. Radiol. 38 (12), 1190–1196. 10.1007/s11604-020-01028-x 32767200

[B7] EASL European Association for the Study of the Liver (2018). EASL clinical practice guidelines: management of hepatocellular carcinoma. J. Hepatol. 69 (1), 182–236. 10.1016/j.jhep.2018.03.019 29628281

[B8] Garin E. Tselikas L. Guiu B. Chalaye J. Edeline J. de Baere T. (2021). Personalised versus standard dosimetry approach of selective internal radiation therapy in patients with locally advanced hepatocellular carcinoma (DOSISPHERE-01): a randomised, multicentre, open-label phase 2 trial. Lancet Gastroenterol. Hepatol. 6 (1), 17–29. 10.1016/s2468-1253(20)30290-9 33166497

[B9] Hassan-Montero Y. De-Moya-Anegón F. Guerrero-Bote V. P. (2022). SCImago Graphica: a new tool for exploring and visually communicating data. Prof. la Inf./Inf. Prof. 31 (5). 10.3145/epi.2022.sep.02

[B10] Hermann A. L. Dieudonné A. Ronot M. Sanchez M. Pereira H. Chatellier G. (2020). Relationship of tumor radiation-absorbed dose to survival and response in hepatocellular carcinoma treated with transarterial radioembolization with (90)Y in the SARAH study. Radiology 296 (3), 673–684. 10.1148/radiol.2020191606 32602828

[B11] Levillain H. Bagni O. Deroose C. M. Dieudonné A. Gnesin S. Grosser O. S. (2021). International recommendations for personalised selective internal radiation therapy of primary and metastatic liver diseases with yttrium-90 resin microspheres. Eur. J. Nucl. Med. Mol. Imaging 48 (5), 1570–1584. 10.1007/s00259-020-05163-5 33433699 PMC8113219

[B12] Liu D. M. Leung T. W. Chow P. K. Ng D. C. Lee R. C. Kim Y. H. (2022). Clinical consensus statement: selective internal radiation therapy with yttrium 90 resin microspheres for hepatocellular carcinoma in Asia. Int. J. Surg. 102, 106094. 10.1016/j.ijsu.2021.106094 35662438

[B13] McGlynn K. A. Petrick J. L. El-Serag H. B. (2021). Epidemiology of hepatocellular carcinoma. Hepatology 73 (Suppl. 1), 4–13. 10.1002/hep.31288 PMC7577946 32319693

[B14] Miao Y. Zhang Y. Yin L. (2018). Trends in hepatocellular carcinoma research from 2008 to 2017: a bibliometric analysis. PeerJ 6, e5477. 10.7717/peerj.5477 30128213 PMC6098682

[B15] Nelson B. J. Pané S. (2023). Delivering drugs with microrobots. Science 382 (6675), 1120–1122. 10.1126/science.adh3073 38060660

[B16] Ricke J. Klümpen H. J. Amthauer H. Bargellini I. Bartenstein P. de Toni E. N. (2019). Impact of combined selective internal radiation therapy and sorafenib on survival in advanced hepatocellular carcinoma. J. Hepatol. 71 (6), 1164–1174. 10.1016/j.jhep.2019.08.006 31421157

[B17] Rumgay H. Arnold M. Ferlay J. Lesi O. Cabasag C. J. Vignat J. (2022). Global burden of primary liver cancer in 2020 and predictions to 2040. J. Hepatol. 77 (6), 1598–1606. 10.1016/j.jhep.2022.08.021 36208844 PMC9670241

[B18] Salem R. (2023). Selective internal radiation therapy using yttrium-90 in early and intermediate hepatocellular carcinoma. Gastroenterol. Hepatol. (N Y) 19 (7), 411–414. 37771621 PMC10524413

[B19] Shi L. Li D. Tong Q. Jia G. Li X. Zhang L. (2023). Silk fibroin-based embolic agent for transhepatic artery embolization with multiple therapeutic potentials. J. Nanobiotechnology 21 (1), 278. 10.1186/s12951-023-02032-9 37598140 PMC10439629

[B20] Sung H. Ferlay J. Siegel R. L. Laversanne M. Soerjomataram I. Jemal A. (2021). Global cancer statistics 2020: GLOBOCAN estimates of incidence and mortality worldwide for 36 cancers in 185 countries. CA Cancer J. Clin. 71 (3), 209–249. 10.3322/caac.21660 33538338

[B21] Synnestvedt M. B. Chen C. Holmes J. H. (2005). CiteSpace II: visualization and knowledge discovery in bibliographic databases. AMIA Annu. Symp. Proc. 2005, 724–728. 16779135 PMC1560567

[B22] van Eck N. J. Waltman L. (2010). Software survey: VOSviewer, a computer program for bibliometric mapping. Scientometrics 84 (2), 523–538. 10.1007/s11192-009-0146-3 20585380 PMC2883932

[B23] Vilgrain V. Pereira H. Assenat E. Guiu B. Ilonca A. D. Pageaux G. P. (2017). Efficacy and safety of selective internal radiotherapy with yttrium-90 resin microspheres compared with sorafenib in locally advanced and inoperable hepatocellular carcinoma (SARAH): an open-label randomised controlled phase 3 trial. Lancet Oncol. 18 (12), 1624–1636. 10.1016/s1470-2045(17)30683-6 29107679

[B24] Vogel A. Meyer T. Sapisochin G. Salem R. Saborowski A. (2022). Hepatocellular carcinoma. Lancet 400 (10360), 1345–1362. 10.1016/s0140-6736(22)01200-4 36084663

[B25] Yan Z. P. Lin G. Zhao H. Y. Dong Y. H. (1993). Yttrium-90 glass microspheres injected via the portal vein. An experimental study. Acta Radiol. 34 (4), 395–398. 10.1080/02841859309173266 8318305

[B26] Zhang N. He X. F. Niu X. K. (2023). Mapping research trends of transarterial chemoembolization for hepatocellular carcinoma from 2012 to 2021: a bibliometric analysis. World J. Methodol. 13 (4), 345–358. 10.5662/wjm.v13.i4.345 37771871 PMC10523245

