# Supplementary material for: A bibliometric analysis of 30 years of research on transarterial radioembolization (TARE) for hepatocellular carcinoma
Source: Front Pharmacol. 2025 Jan 6;15:1449722. doi: 10.3389/fphar.2024.1449722 (PMC11743607; doi:10.3389/fphar.2024.1449722)
Supplement: Supplementary file 1 [file DataSheet1.docx]

Supplementary Material

# Supplementary Figures

*
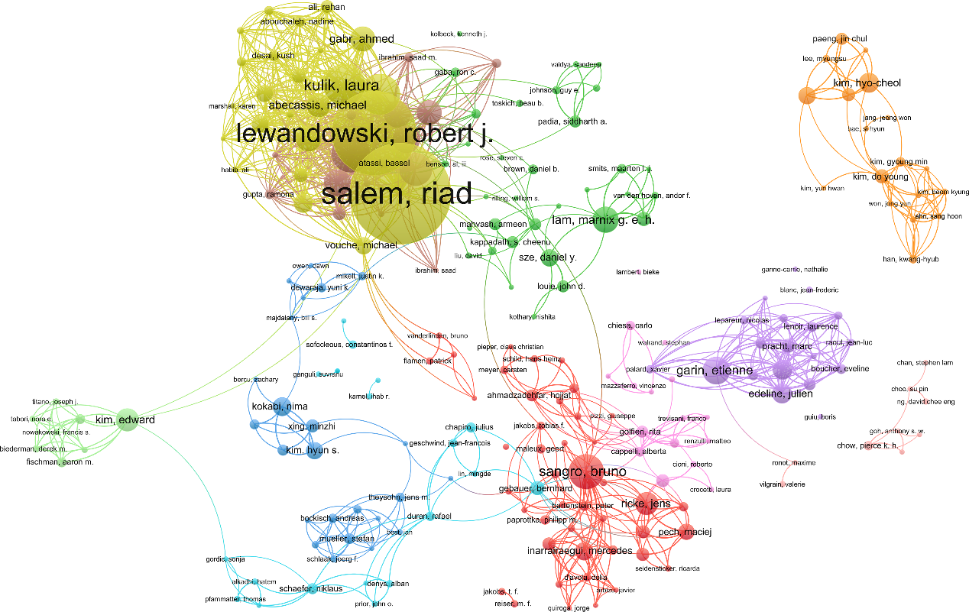
*

**Figure S1**. The co-authorship analysis of Author collaboration.


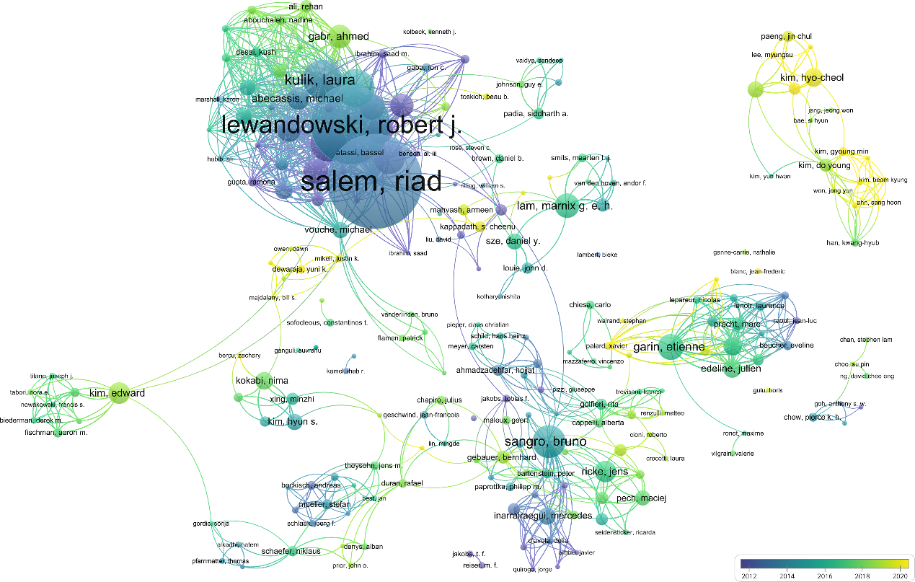


**Figure S2.** The time-overlapping map co-authorship analysis of Author collaboration.
